# No effect of test and treat on sexual behaviours at population level in rural South Africa

**DOI:** 10.1097/QAD.0000000000002104

**Published:** 2019-01-03

**Authors:** Matthieu Rolland, Nuala McGrath, Thierry Tiendrebeogo, Joseph Larmarange, Deenan Pillay, François Dabis, Joanna Orne-Gliemann

**Affiliations:** a University Bordeaux, ISPED, Inserm, Bordeaux Population Health Research Center, UMR 1219, Bordeaux, France; b Academic Unit of Primary Care and Population Sciences and Department of Social Statistics and Demography, University of Southampton, Southampton, UK; c Africa Health Research Institute, Somkhele, Mtubatuba; d School of Nursing & Public Health, University of KwaZulu-Natal, KwaZulu-Natal, South Africa; e Research Department of Epidemiology & Public Health, University College London, London, UK; f Centre Population et Développement, Institut de Recherche pour le Développement, Université Paris Descartes, Inserm, Paris, France; g Division of Infection and Immunity, University College London, London, UK.

**Keywords:** Africa, antiretroviral therapy, HIV, prevention, sexual behaviour

## Abstract

**Context:**

Within the community-randomized ANRS 12249 Treatment-as-Prevention trial conducted in rural South Africa, we analysed sexual behaviours stratified by sex over time, comparing immediate antiretroviral therapy irrespective of CD4^+^ cell count vs. CD4^+^-guided antiretroviral therapy (start at CD4^+^ cell count > 350 cells/μl then >500 cells/μl) arms.

**Methods:**

As part of the 6-monthly home-based trial rounds, a sexual behaviour individual questionnaire was administered to all residents at least 16 years. We considered seven indicators: sexual intercourse in the past month; at least one regular sexual partner in the past 6 months; at least one casual sexual partner in the past 6 months and more than one sexual partner in the past 6 months; condom use at last sex (CLS) with regular partner, CLS with casual partner, and point prevalence estimate of concurrency. We conducted repeated cross-sectional analyses, stratified by sex. Generalized Estimating Equations models were used, including trial arm, trial time, calendar time and interaction between trial arm and trial time.

**Results:**

CLS with regular partner varied between 29–51% and 23–46% for men and women, respectively, with significantly lower odds among women in the control vs. intervention arm by trial end (*P* < 0.001). CLS with casual partner among men showed a significant interaction between arm and trial round, with no consistent pattern. Women declared more than one partner in the past 6 months in less than 1% of individual questionnaires; among men, rates varied between 5–12%, and odds significantly and continuously declined between calendar rounds 1 and 7 [odds ratio = 4.2 (3.24–5.45)].

**Conclusion:**

Universal Test and Treat was not associated with increased sexual risk behaviours.

## Introduction

Universal antiretroviral therapy (ART) at high CD4^+^ cell counts reduces morbidity and mortality rates among people living with HIV [1,2] and reduces the risk of transmission to HIV-negative partners [3]. Over the past few years, following the 2015 WHO ART initiation guidelines [4] and the repeated calls to improve ART coverage [5], implementation of universal ART is being generalized. Mathematical models suggested that important reductions in HIV transmission were achievable with universal test and treat (UTT), that is high uptake of regular HIV testing and universal ART initiation when diagnosed HIV-positive [6,7].

One of the premises of UTT strategies is that ART decreases infectivity. However, there has been concern that increased access to ART could alter HIV risk perception, and lead to sexual disinhibition or risk compensation [8], which could potentiate the continued spread of the epidemic.

There is no evidence of increased sexual risk behaviours related to increased access to ART among high-risk populations in high-resource settings [9]. In sub-Saharan Africa, where HIV epidemics are largely driven by heterosexual transmission, available data among people living with HIV on ART does not suggest any increase in at-risk sexual behaviours and no increase in partner acquisition or partnership dissolution [10,11]. Studies conducted in the context of early or universal ART did not report any increase in rates of condom-less sex over time [12] and sexual behaviours did not differ between HIV-infected people treated below and above the 350 cells/μl CD4^+^ cell count threshold [13]. Among sero-discordant couples followed within the Partners-PrEP study in Eastern Africa, ART was associated with a significant decrease in reports of condom-less vaginal sex acts with HIV-uninfected partners [14]. Recent work conducted in rural KwaZulu Natal (KZN) province (South Africa) found no evidence of increased sexual risk-taking at the population level following ART availability, and even protective changes in some behaviours [15].

The consequences of UTT roll-out on sexual behaviours at population-level are still largely unknown. Our primary objective was to assess the impact of universal ART on sexual behaviours at population-level in the context of the ANRS 12249 Treatment-as-Prevention (TasP) study conducted in rural KZN. Our secondary objective was to analyse the change over time of sexual behaviours in the study area.

## Methods

### Treatment-as-Prevention trial design and setting

The TasP study is a cluster-randomized trial conducted by the Africa Health Research Institute (AHRI) that investigated whether UTT reduces HIV incidence at population-level [16,17]. It was implemented in Hlabisa subdistrict, northeast KZN, in a largely rural area, with approximately 28 000 resident adults, and an HIV prevalence reaching 30% [18]. It was approved by the Biomedical Research Ethics Committee, University of KZN (BFC 104/11) and the Medicines Control Council of South Africa (ClinicalTrials.gov: NCT01509508; South African National Clinical Trials Register: DOH-27-0512-3974). Follow-up began in four clusters in 2012, was expanded to 10 clusters in 2013, and from 2014 the trial included 22 clusters (11 × 2) (Supplementary data, Fig. 1). The TasP study did not show any significant population-level impact of universal ART (vs. national ART initiation guidelines) on cumulative HIV incidence [19].

### Study procedures

Six-monthly home-based survey rounds were implemented, during which all eligible individuals (aged 16 years and older, resident in the trial area) were enumerated and given a unique identifier; the list of household members was updated to take into account deaths, out-migration, in-migration and individuals reaching the age of 16 years; after written informed consent, participants were offered point-of-care rapid HIV counselling and testing. In the intervention clusters, HIV-positive participants were offered ART regardless of CD4^+^ cell count, whereas in the control clusters, ART was provided according to national guidelines (initially CD4^+^ cell count ≤ 350 cells/μl, then <500 cells/μl from January 2015).

### Socio-demographic and sexual behaviours questionnaire

TasP trial participants were invited to respond to a socio-demographic and sexual behaviours questionnaire (individual questionnaire), administered face-to-face by fieldworkers/HIV counsellors as part of the home-based survey rounds. It was based on items used in previous AHRI research studies [15]. Sexual behaviour data described the type, duration and sexual risks associated with up to the three most recent sexual partnerships of participants in the last 12 months. For those with no partner in the last 12 months, details of the most recent partner were recorded.

### Outcomes

Five indicators were computed at the level of the participant: sexual intercourse in the past month, at least one regular sexual partner reported in the past 6 months, at least one casual sexual partner reported in the past 6 months, at least two sexual partners reported in the past 6 months, and point prevalence estimate of concurrency [20]. Two indicators were computed at the partnership level: condom use at last sex (CLS) with regular partner, CLS with casual partner. For participant-level indicators each individual questionnaire contributed one observation that took into account information from all the reported partnerships for the past 6 months. For partnership-level indicators each individual questionnaire contributed as many observations as there were partnerships reported (i.e. between 0 and 3). To avoid duplicates due to the individual questionnaire being administered every 6 months, partnerships with date of last sex missing or more than 6 months were not included in our analyses.

### Statistical analysis

We conducted a repeated cross-sectional analysis of sexual behaviours among the resident population, regardless of whether participants had participated to the previous round or the next round: each survey round was considered as a cross-sectional survey.

To estimate indicators representative of the entire population, and to account for varying participation rates per cluster and survey round, we adjusted estimates using poststratification weights [21] that accounted for the distribution of sex, age group, education level, professional status and marital status of the eligible population, separately by cluster, for each survey round. For participants whose characteristics were not documented at a given survey round, we used the closest available questionnaire. In the rare cases in which a participant characteristic was not documented at any point, multi-factorial analysis was used to impute missing socio-demographic with the imputeFAMD method of R's missMDA package [22]. In the rare cases in which people completed two questionnaires in the same survey round, half the weight was applied to each questionnaire. No sexual behaviour data was collected from trial participants from the clusters opened in 2012 (*n* = 4 clusters) and 2013 (*n* = 6 clusters) during their second and third survey round, respectively. These data were considered as missing data.

Because the clusters were included in different stages, we included in our analyses two different time scales to distinguish contextual time trends from intervention-related time trends. Calendar time relates to the overall number of survey rounds implemented since the start of the study in March 2012 (expressed in calendar rounds). Trial time relates to the number of survey rounds implemented from the date each cluster was included in the study (expressed in trial rounds and starting respectively in March 2012 for the first series of clusters, January 2013 for the second and June 2014 for the last). For example for a cluster that was in the group of clusters opened in January 2013, the second round of questionnaires was at Trial Round 2, which corresponds to the Calendar Round 3 (Table 1).

**Table 1:**
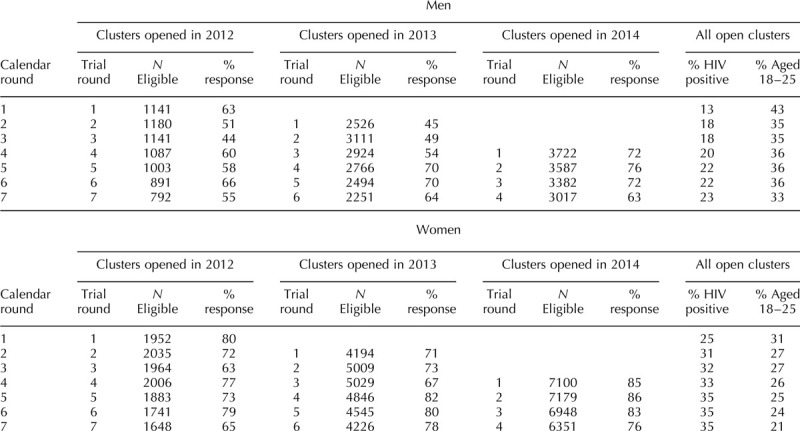
Number of individuals eligible to answer the individual questionnaire and response rate per calendar round, stratified by sex and cluster group. ANRS 12249 TasP trial (2012–2016).

**Fig. 1:**
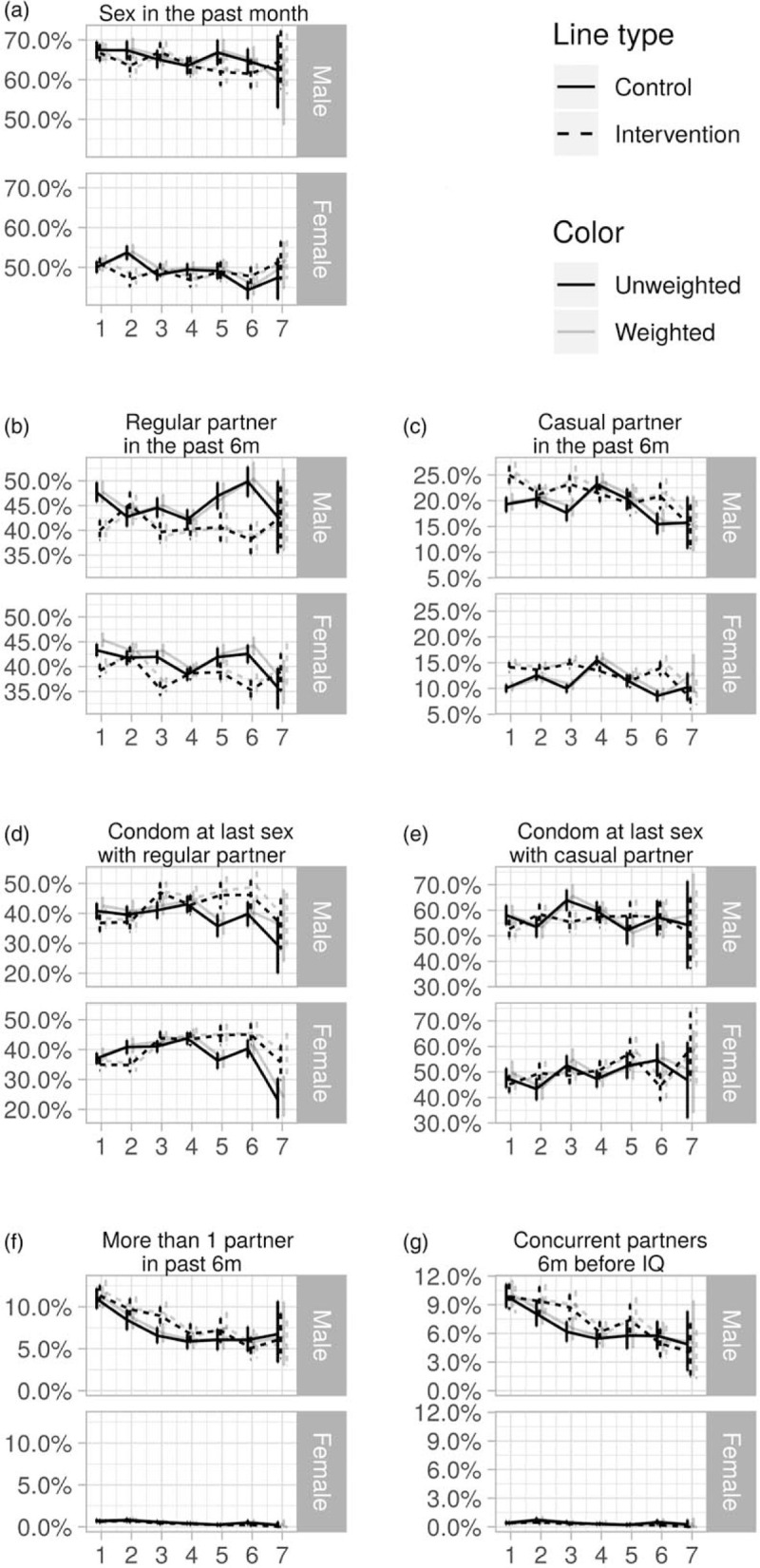
Unweighted and weighted sexual behaviour indicators in rural KwaZulu-Natal, by trial rounds, sex and trial arm with 95% confidence intervals, ANRS 12249 TasP trial (2012–2016).

We first described the proportion for each indicator by trial round, sex and trial arm, with and without survey weights (Fig. 1). The 95% confidence intervals were computed using the 2.5 and 97.5 percentile of the bootstrapped distribution of the proportion using the boot function of R's boot package [23].

We then computed multivariate models for each indicator, separately for each sex and using post-stratification weights. To account for multiple observations per participant, marginal Generalized Estimating Equations models of logistic regression assuming an independent correlation structure [24] were used, using the R package geepack [25]. First, a full model was computed for each indicator. This model included *trial arm*, which represents the baseline difference between arms; *trial round,* which represents the effect over time of the trial interventions implemented in both arms; an *interaction term* between trial arm and trial rounds, which represents the specific effect over time of universal ART implemented in the intervention arm only; and *calendar round,* which represents the structural change over time in the study area (independently of trial implementation). Trial rounds and calendar rounds were represented by dummy indicators to allow for non-linear trends (refer to Supplementary data, Table S1). Once the full model was computed, the most parsimonious model with the lowest Quasi-Akaike Criterion was kept. This was evaluated using the dredge function of R's MuMin package [26]. In all cases, trial arm remained in the final model to allow estimation of the association between trial arm and each outcome. To visualize possible patterns, when the interaction between arm and trial round was selected in the final model, the model was re-run with arm and trial round combinations represented directly by dummy variables. All analyses were performed using R version 3.3.2 [27].

## Results

### Study population

Overall during the study period, participation rate at each survey round varied between 45 and 76% for men and between 71 and 86% for women (Table 1). A total of 9008 men and 16 672 women were included in the study. 66 120 partnerships were reported. Among those, 10 564 partnerships were excluded due to missing date of last sex and 12 957 due to last sex being more than 6 months prior to individual questionnaire. Finally, a total of 15 831 partnerships reported by men (10 199 regular and 5632 casual) and 26 460 by women (20 083 regular and 6377 casual) were included.

### Differences in sexual behaviours

Figure 1 presents the weighted and unweighted proportions for men and women, by trial arm and trial round, for the seven sexual behaviour indicators. For all indicators applying weights did not significantly change the estimates (Fig. 1) and for all remaining analyses weighted estimates are presented. The results of the final model for each indicator are presented in Tables 2–4 (men) and Tables 5 and 6 (women). Interaction model outputs (Table 7) are presented visually in Supplementary data (Fig. 3).

**Table 2:**
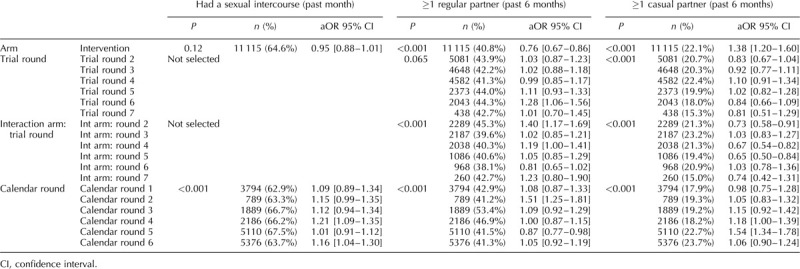
Final multivariable model for each sexual behaviour indicator among men in the ANRS 12249 TasP trial (2012–2016).

**Table 3:**
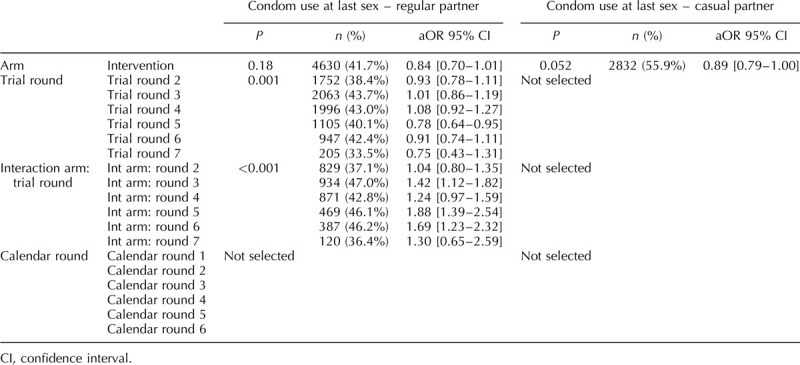
Final multivariable model for each sexual behaviour indicator among men in the ANRS 12249 TasP trial (2012–2016).

**Table 4:**
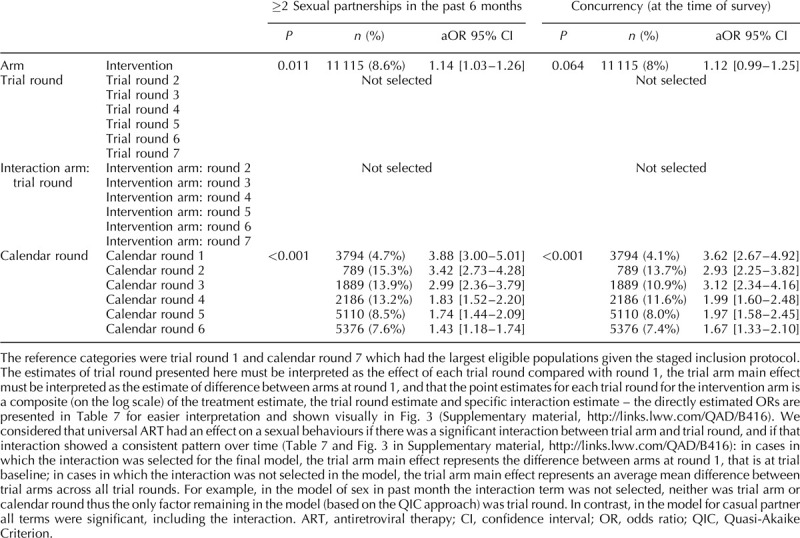
Final multivariable model for each sexual behaviour indicator among men in the ANRS 12249 TasP trial (2012–2016).

**Table 5:**
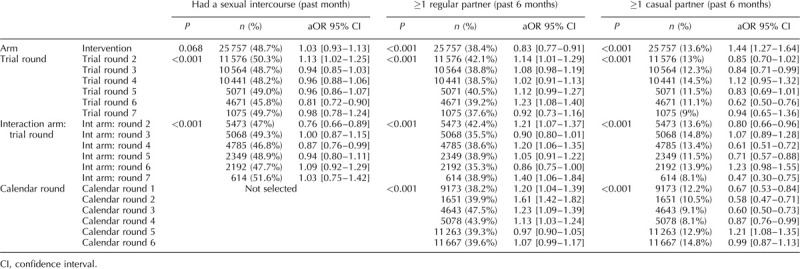
Final multivariable model for each sexual behaviour indicator among women in the ANRS 12249 TasP trial (2012–2016).

**Table 6:**
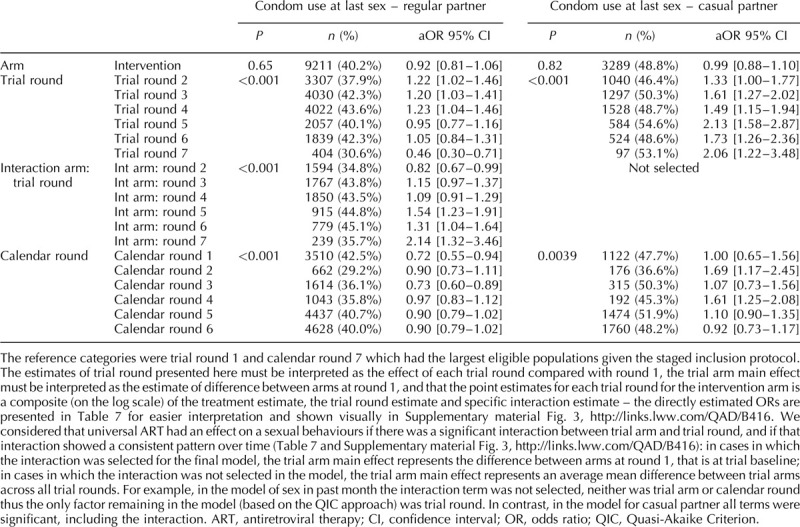
Final multivariable model for each sexual behaviour indicator among women in the ANRS 12249 TasP trial (2012–2016).

**Table 7:**
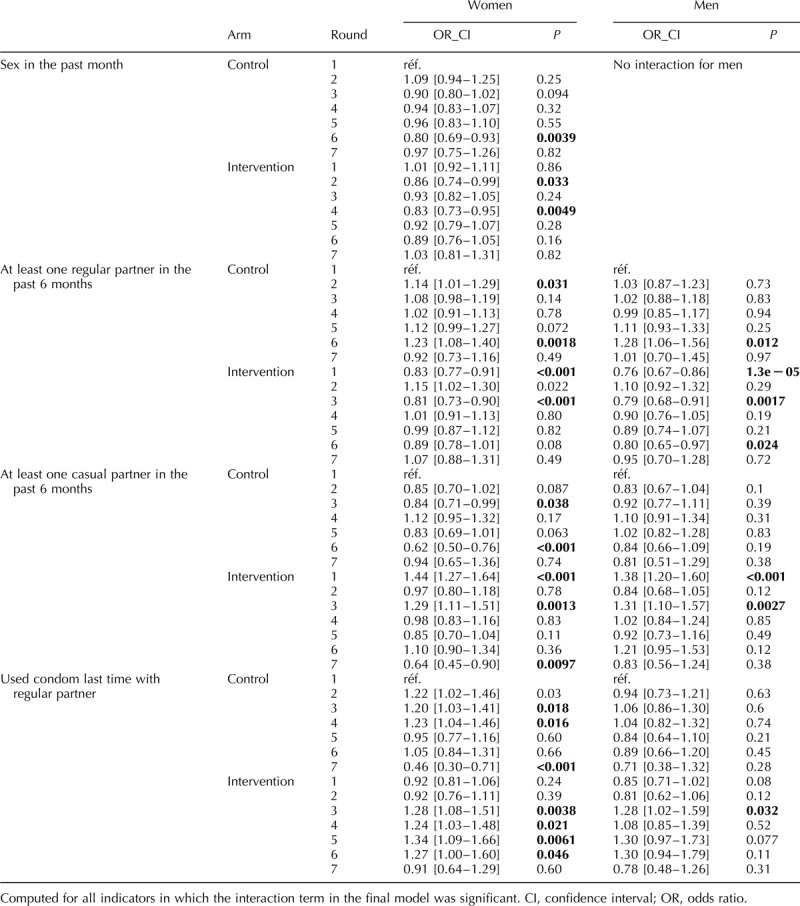
Model output with arm and trial round combinations represented directly by dummy variables (ANRS 12249 TasP trial (2012–2016).

### Sexual intercourse in the past month

**Fig. 2:**
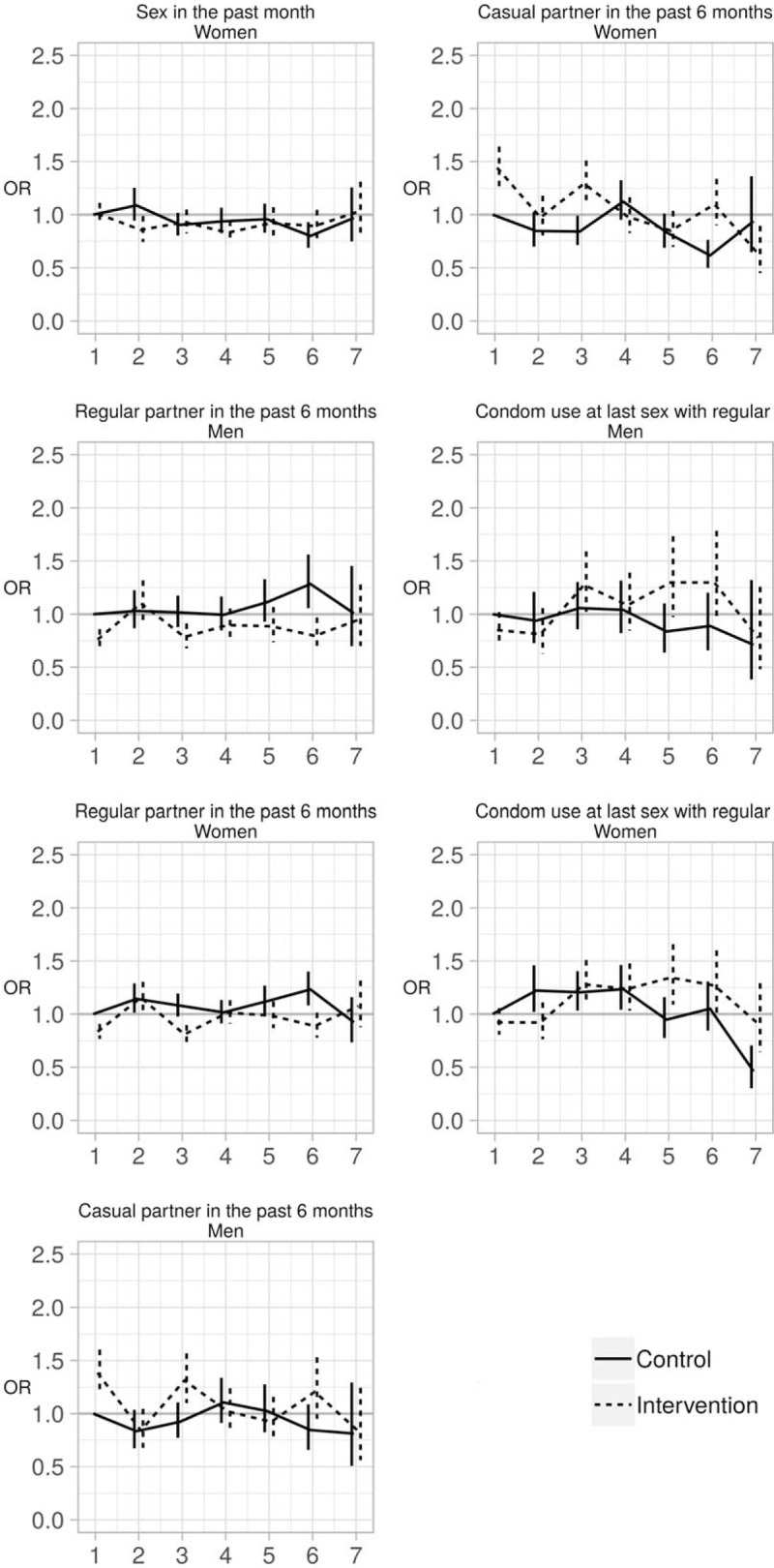
Odds ratios and 95% confidence intervals plots of the model output with arm and trial round combinations represented directly by dummy variables.

*Note:* Computed for all indicators in which the interaction term in the final model was significant

The proportion of individual questionnaires at which sexual intercourse was reported in the past month varied between 59 and 68% for men and between 44 and 54% for women (Fig. 1a). Among men, there was no difference according to arm. Among women, the interaction between arm and trial round was significant (*P* < 0.001; Tables 5 and 6). The odds of women reporting sexual intercourse showed significant variations between arms across trial rounds but with no consistent pattern (Fig. 2, Table 7).

### Regular partner in the past 6 months

The proportion of individual questionnaires at which a regular partner in the past 6 months was declared varied between 38 and 50% for men and between 35 and 46% for women (Fig. 1b). There was a significant interaction between arm and trial round for men and women (both: *P* < 0.001; Tables 2–6) with statistically significantly lower odds of reporting a regular partner in the intervention arm compared with the control arm for both sexes at enrolment, and no consistent pattern over trial rounds (Fig. 2, Table 7). For both men and women, the odds of reporting a regular partner varied significantly across calendar rounds, with no consistent pattern (Tables 2–6).

### Casual partner in the past 6 months

The proportion of individual questionnaires at which a casual partner in the past 6 months was declared varied between 15 and 26% for men and between 8 and 17% for women (Fig. 1c). There was a significant interaction between arm and trial round for men and women (both: *P* < 0.001; Tables 2–6), with statistically significantly higher odds of reporting a casual partner in the intervention arm compared with the control arm for both sexes at enrolment, and no consistent pattern over trial rounds. Figure 1c and Table 7 suggest that for women, the odds of reporting a casual partner in the past 6 months decreased more rapidly in the intervention arm than in the control arm between trial rounds 1 and 7. At round 1, the odds are significantly higher in the intervention arm than in the control arm [odds ratio (OR) = 1.46 (1.29–1.67), *P* < 0.001, Table 7]. At round 7, the odds in the intervention arm were significantly lower than the reference category [OR = 0.64 (0.46–0.91), *P* = 0.012, Table 7], whereas the odds in the control arm were not (*P* = 0.8, Table 7). The odds of reporting a casual partner among men and women significantly varied across calendar round, with no consistent pattern (Tables 2–6, Fig. 2).

### Condom use at last sex with regular partner

CLS with a regular partner for men varied between 29 and 51% and between 23 and 46% for women (Fig. 1d). There was a significant interaction between arm and trial round for men and women (both: *P* < 0.001; Tables 2–6), with no consistent pattern over trial rounds for men (Table 7). For women, Table 7 suggests that the odds of reporting CLS with a regular partner decreased more rapidly in the control arm than in the intervention arm between trial rounds 1 and 7. At round 1, the odds were not significantly different (*P* = 0.2, Table 7). At round 7, the odds in the control arm were significantly lower than the reference category [OR = 0.46 (0.30–0.70), *P* < 0.001, Table 4], whereas the odds in the intervention arm were not (*P* = 0.56, Table 7). For men there was no significant variation in CLS with a regular partner over calendar round (Tables 2–4). For women the odds of reporting CLS with a regular partner varied significantly across calendar round with no consistent pattern (Fig. 2, Tables 5 and 6).

### Condom use at last sex with casual partner

CLS with a casual partner varied between 51 and 65% for men and between 43 and 60% for women (Fig. 1e). There was no significant differences between arms (Tables 2–4). However for women, there seems to have been an increase between trial round 1 and 7 [OR = 2.03 (1.21–3.43), *P* < 0.001; Tables 5 and 6]. Among women the odds of reporting CLS with a casual partner varied significantly across calendar round with no consistent pattern (Tables 5 and 6).

### Two or more sexual partners in the past 6 months

The percentage of individual questionnaires at which men declared more than one partner in the past 6 months varied between 5 and 12% and remained below 1% for women (Fig. 1f). Hence, only the model for men was produced. The interaction between arm and trial round was not statistically significant. Overall the odds of men reporting two or more sexual partners in the past 6 months were higher in the intervention arm compared with the control arm [OR = 1.15 (0.03–1.28), *P* = 0.015; Tables 2–4]. The odds of men reporting two or more sexual partners in the past 6 months significantly and continuously declined between calendar rounds 1 and 7 [OR = 4.2 (3.24–5.45)] (Tables 2–4).

### Concurrency

The percentage of individual questionnaires at which men declared concurrent relationships varied between 4 and 11% and remained below 1% for women (Fig. 1g). The interaction between arm and trial round was not statistically significant. Concurrency among men significantly and continuously declined between calendar rounds 1 and 7 (Tables 2–4).

## Discussion

In this study, we reported on the impact of universal ART on sexual behaviours at population-level in rural South Africa. Virtually no signs of sustained changes in sexual behaviour were observed in the universal ART arm compared with the standard guidelines arm, and in particular sexual disinhibition was not observed for any of the indicators under study.

At the start of the trial, men and women in the intervention arm were more likely to report casual partners and less likely to report regular partners than in the control arm. Men in the intervention arm were also more likely to report more than one partner in the last 6 months than in the control arm.

Proportions of sexual intercourse in the past month among women, regular partner among women, regular and casual partner among men and CLS with regular partners among men fluctuated over time. This may be related to changes in trial participation rate and changes in the population profile inherent to repeated cross-sectional survey designs. We used sampling weights to adjust for differences in participation rates. Further, mobility in this rural population is high, with an estimated 10–20% population change at each survey round due to in-out migration [28]. In addition, the high numbers of questionnaires (>75 000) provided sufficient power for very small differences in the prevalence of sexual behaviour indicators to be statistically significant.

For two indicators, reporting a casual partner in the past 6 months among women and CLS with a regular partner among women, the interaction between arm and trial round was significant with signs of consistent diverging trends between the two arms between trial rounds 1 and 7. The odds of casual partners seem to have decreased in the intervention arm compared with the control arm and the rates of condom use with a regular partner at last sex seem to have increased in the intervention compared with the control arm. These differences may be reflect the fact that round 7 only includes four clusters out of the total 22, but in any case it would suggest a beneficial effect of messaging around universal ART on condom use. This finding would confirm the trends in reduced unprotected sex documented in South Africa among HIV-infected clinic attendees after ART initiation [10], or in Uganda among HIV-negative household members living with HIV-infected people initiated on ART [29].

Two indicators showed clear improvements over time. The odds of women declaring having used a condom at last sex with a casual partner was significantly higher in the later trial rounds than in the first trial round (Tables 2–4). This may be a result of the repeated exposure to trial staff and preventive messages, and to increased risk perception associated with unprotected sexual intercourse. And finally we observed a decrease over calendar time in the proportion of men reporting two or more sexual partners in the past 6 months and of concurrency. This time trend is however different from what was found in a recent meta-analysis, in which reporting multiple partners over the past 12 months increased consistently across almost all study countries [30].

There are several limits to our analysis. First, the phased approach of the TasP trial may have biased the population characteristics, as the clusters were not opened at random. This can mainly be observed at rounds in which only the first set of clusters are under observation (calendar round 1 and trial round 7); significant differences at these rounds thus needed to be treated carefully and were actually not considered sufficient to argue for an arm difference. Participation rates changed over time and were not uniform within the population. However, we observed no significant difference between the weighted and unweighted results. Second, the collection of sexual behaviour data was not homogenous over time, resulting in missing data for calendar rounds 2 and 3. Third, we chose to retain the most parsimonious model to preserve power for our primary question: analysing differences between arms during follow-up for each sexual behaviour indicator. Although parsimonious models do not allow direct comparison between the different indicators, they ensure that the question is answered in the ‘best’ (most parsimonious) way for each indicator, even while using the same approach for all indicators. The full model results are presented in Tables S2 and S3. For sexual intercourse in the past month among women and CLS with a casual partner for men and women, the significant time variables are not the same as for the parsimonious models. However, the added significant variables show no trend. And the arm difference for concurrency among men, which was close to significance in the parsimonious model (*P* = 0.064), becomes significant in the full model. Thus interpretation for these indicators did not change substantially between the full and parsimonious models. Forth, our analysis is based on self-reported data. The changes we observed over time may thus reflect changes in reporting sexual behaviours rather changes in sexual behaviours per se. Finally, we cannot assume that the TasP population is representative of rural KZN or even of all of Hlabisa subdistrict.

The TasP trial was unable to show any evidence of universal ART on the prevention of HIV transmission; with HIV incidence remaining comparable between both trial arms and population-level increase in ART coverage being similar in both arms [19]. We did not observe any signs of sexual disinhibition associated with early ART in this rural population of KZN. The lack of difference in HIV incidence between both arms cannot be explained by increased sexual risk taking in the intervention arm. Continued monitoring of population-level sexual behaviour indicators is needed as the UTT strategy is rolled out. In particular multiple partnerships, partnership selection/dissolution, sero-sorting or ‘ART-sorting’ phenomenon, which may also affect in the end the impact of ART on HIV incidence [31].

## Acknowledgements

We thank the study volunteers for allowing us into their homes and participating in this trial, the KwaZulu-Natal Provincial and the National Department of Health of South Africa for their support of this study.

We thank staff of the Africa Health Research Institute (previously Africa Centre for Population Health) for the trial implementation and analysis of data, including those who conducted the fieldwork, provided clinical care, developed and maintained the database, entered the data and verified data quality.

We acknowledge the advice and support of the Scientific Advisory Board: Bernard Hirschel (Chair), Xavier Anglaret, Hoosen Coovadia, Eric Djimeu, Eric Fleutelot, Eric Goemaere, Alice Jacob, Jean-Michel Molina, Lynn Morris, Golriz Pahlavan-Grumel, Calice Talom, Francois Venter, Sibongile Zungu.

We acknowledge the advice and support of the DSMB: Patrick Yeni (Chair), Nathan Ford, Hakima Himmich, Catherine Hankins, Helen Weiss, Sinead Delany-Moretlwe.

Special thanks to Professor Jean-François Delfraissy, prior Director of ANRS.

Research discussed in this publication has been cofunded by the ANRS, the Deutsche Gesellschaft für Internationale Zusammenarbeit (GIZ), and the International Initiative for Impact Evaluation, Inc. (3ie) with support from the Bill & Melinda Gates Foundation. The content is solely the responsibility of the authors and does not represent the official views of the ANRS, 3ie or the Bill & Melinda Gates Foundation.

The trial was conducted with the support of Merck & Co. Inc and Gilead Sciences that provided the Atripla drug supply. The Africa Health Research Institute receives core funding from the Wellcome Trust, which provides the platform for the population-based and clinic-based research at the Centre.

### Conflicts of interest

There are no conflicts of interest.

## Supplementary Material

Supplemental Digital Content
