# Supplementary material for: No effect of test and treat on sexual behaviours at population level in rural South Africa
Source: AIDS. 2019 Jan 3;33(4):709–22. doi: 10.1097/QAD.0000000000002104 (PMC7373441; doi:10.1097/QAD.0000000000002104)
Supplement: Supplemental Digital Content [file aids-33-709-s001.docx]

**Supplementary material – ROLLAND et al**

**Figure S1.** Time scales used in the ANRS 12249 TasP trial (2012-2016): trial round vs calendar round for each cluster group.


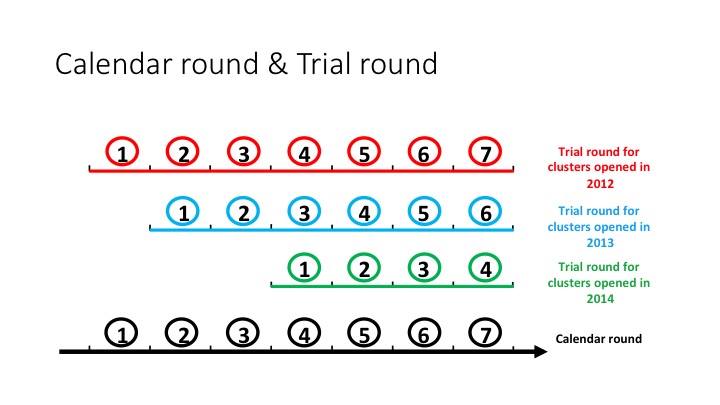


**Table S1.** Multivariable model output for each sexual behaviour indicator in the ANRS 12249 TasP trial (2012-2016) with arm and trial round combinations represented directly by dummy variables.

|  |  |  | **Women** | | **Men** | |
| --- | --- | --- | --- | --- | --- | --- |
|  | **Arm** | **Round** | **OR_CI** | **pval** | **OR_CI** | **pval** |
| **Sex in the past month** | **Control** | 1 | Réf. |  | Not interaction for men | |
|  |  | 2 | 1.09 [0.94 - 1.25] | 0.30 |  |  |
|  |  | 3 | 0.90 [0.80 - 1.02] | 0.09 |  |  |
|  |  | 4 | 0.94 [0.83 - 1.07] | 0.32 |  |  |
|  |  | 5 | 0.96 [0.83 - 1.11] | 0.55 |  |  |
|  |  | 6 | 0.80 [0.69 - 0.93] | **0.0039** |  |  |
|  |  | 7 | 0.97 [0.75 - 1.25] | 0.82 |  |  |
|  | **Intervention** | 1 | 1.01 [0.92 - 1.12] | 0.86 |  |  |
|  |  | 2 | 0.86 [0.74 - 0.99] | **0.033** |  |  |
|  |  | 3 | 0.93 [0.82 - 1.05] | 0.24 |  |  |
|  |  | 4 | 0.83 [0.73 - 0.95] | **0.0049** |  |  |
|  |  | 5 | 0.92 [0.79 - 1.07] | 0.28 |  |  |
|  |  | 6 | 0.89 [0.76 - 1.05] | 0.16 |  |  |
|  |  | 7 | 1.03 [0.81 - 1.31] | 0.82 |  |  |
| **Regular partner in the past 6 months** | **Control** | 1 | Réf. | Réf. |  |  |
|  |  | 2 | 1.14 [1.01 - 1.29] | **0.031** | 1.03 [0.87 - 1.23] | 0.73 |
|  |  | 3 | 1.08 [0.98 - 1.19] | 0.14 | 1.02 [0.88 - 1.18] | 0.83 |
|  |  | 4 | 1.02 [0.91 - 1.13] | 0.78 | 0.99 [0.85 - 1.17] | 0.94 |
|  |  | 5 | 1.12 [0.99 - 1.27] | 0.072 | 1.11 [0.93 - 1.33] | 0.25 |
|  |  | 6 | 1.23 [1.08 - 1.40] | **0.0018** | 1.29 [1.06 - 1.56] | **0.012** |
|  |  | 7 | 0.92 [0.73 - 1.16] | 0.49 | 1.01 [0.70 - 1.45] | 0.97 |
|  | **Intervention** | 1 | 0.83 [0.76 - 0.91] | **<0.001** | 0.76 [0.67 - 0.86] | **<0.001** |
|  |  | 2 | 1.15 [1.02 - 1.31] | **0.022** | 1.10 [0.92 - 1.32] | 0.29 |
|  |  | 3 | 0.81 [0.73 - 0.90] | **<0.001** | 0.79 [0.68 - 0.91] | **0.0017** |
|  |  | 4 | 1.02 [0.91 - 1.13] | 0.80 | 0.90 [0.76 - 1.05] | 0.19 |
|  |  | 5 | 0.99 [0.87 - 1.12] | 0.82 | 0.89 [0.74 - 1.07] | 0.26 |
|  |  | 6 | 0.89 [0.78 - 1.01] | 0.086 | 0.80 [0.65 - 0.97] | **0.024** |
|  |  | 7 | 1.08 [0.88 - 1.31] | 0.49 | 0.95 [0.70 - 1.28] | 0.72 |
| **Casual partner in the past 6 months** | **Control** | 1 | Réf. | Réf. |  |  |
|  |  | 2 | 0.85 [0.70 - 1.02] | 0.087 | 0.83 [0.67 - 1.04] | 0.10 |
|  |  | 3 | 0.84 [0.71 - 0.99] | **0.038** | 0.92 [0.77 - 1.11] | 0.39 |
|  |  | 4 | 1.12 [0.95 - 1.32] | 0.17 | 1.10 [0.91 - 1.34] | 0.31 |
|  |  | 5 | 0.83 [0.69 - 1.01] | 0.063 | 1.02 [0.82 - 1.28] | 0.83 |
|  |  | 6 | 0.62 [0.50 - 0.76] | **<0.001** | 0.84 [0.66 - 1.09] | 0.19 |
|  |  | 7 | 0.94 [0.65 - 1.36] | 0.74 | 0.81 [0.51 - 1.29] | 0.38 |
|  | **Intervention** | 1 | 1.44 [1.27 - 1.64] | **<0.001** | 1.38 [1.20 - 1.60] | **<0.001** |
|  |  | 2 | 0.97 [0.80 - 1.18] | 0.78 | 0.84 [0.68 - 1.05] | 0.12 |
|  |  | 3 | 1.29 [1.11 - 1.51] | **0.0013** | 1.31 [1.10 - 1.57] | **0.0027** |
|  |  | 4 | 0.98 [0.83 - 1.16] | 0.83 | 1.02 [0.84 - 1.24] | 0.85 |
|  |  | 5 | 0.85 [0.70 - 1.04] | 0.11 | 0.92 [0.73 - 1.16] | 0.49 |
|  |  | 6 | 1.10 [0.90 - 1.34] | 0.36 | 1.21 [0.95 - 1.53] | 0.12 |
|  |  | 7 | 0.64 [0.45 - 0.90] | **0.0097** | 0.83 [0.56 - 1.24] | 0.38 |
| **Condom use at last sex with a regular partner** | **Control** | 1 | Réf. | Réf. |  |  |
|  |  | 2 | 1.22 [1.02 - 1.46] | 0.03 | 0.94 [0.73 - 1.21] | 0.63 |
|  |  | 3 | 1.20 [1.03 - 1.41] | **0.018** | 1.06 [0.86 - 1.30] | 0.60 |
|  |  | 4 | 1.23 [1.04 - 1.46] | **0.016** | 1.04 [0.82 - 1.32] | 0.74 |
|  |  | 5 | 0.95 [0.77 - 1.16] | 0.60 | 0.84 [0.64 - 1.10] | 0.21 |
|  |  | 6 | 1.05 [0.84 - 1.31] | 0.66 | 0.89 [0.66 - 1.20] | 0.45 |
|  |  | 7 | 0.46 [0.30 - 0.71] | **<0.001** | 0.71 [0.38 - 1.32] | 0.28 |
|  | **Intervention** | 1 | 0.92 [0.81 - 1.06] | 0.24 | 0.87 [0.72 - 1.05] | 0.14 |
|  |  | 2 | 0.92 [0.76 - 1.11] | 0.39 | 0.85 [0.65 - 1.11] | 0.23 |
|  |  | 3 | 1.28 [1.08 - 1.51] | **0.0038** | 1.33 [1.07 - 1.67] | **0.012** |
|  |  | 4 | 1.24 [1.03 - 1.48] | **0.021** | 1.17 [0.91 - 1.50] | 0.23 |
|  |  | 5 | 1.34 [1.09 - 1.66] | **0.0061** | 1.38 [1.03 - 1.85] | **0.031** |
|  |  | 6 | 1.27 [1.00 - 1.60] | **0.046** | 1.37 [1.00 - 1.90] | 0.052 |
|  |  | 7 | 0.91 [0.64 - 1.29] | 0.60 | 0.83 [0.51 - 1.35] | 0.45 |

*Note: Computed for all indicators where the interaction term in the final model was significant*

**Table S2.** Full multivariable model for each sexual behaviour indicator among men in the ANRS 12249 TasP trial (2012-2016) (1/3)

|  |  | **Had a sexual intercourse (past month)** | | | **≥ 1 regular partner (past 6 months)** | | | **≥ 1 casual Partner (past 6 months)** | | |  |
| --- | --- | --- | --- | --- | --- | --- | --- | --- | --- | --- | --- |
|  |  | **pval** | **n (%)** | **aOR 95CI** | **pval** | **n (%)** | **aOR 95CI** | **pval** | **n (%)** | **aOR 95CI** | |
| **Arm** | Intervention | 0.12 | 11115 (64.6%) | 0.98 [0.84 - 1.14] | <0.001 | 11115 (40.8%) | 0.76 [0.67 - 0.86] | <0.001 | 11115 (22.1%) | 1.38 [1.2 - 1.6] | |
| **Trial Round** | Trial round 2 | 0.47 | 5081 (65.5%) | 1.17 [0.94 - 1.46] | 0.073 | 5081 (43.9%) | 1.03 [0.87 - 1.23] | <0.001 | 5081 (20.7%) | 0.83 [0.67 - 1.04] | |
|  | Trial round 3 |  | 4648 (66.0%) | 0.91 [0.76 - 1.10] |  | 4648 (42.2%) | 1.02 [0.88 - 1.18] |  | 4648 (20.3%) | 0.92 [0.77 - 1.11] | |
|  | Trial round 4 |  | 4582 (63.4%) | 0.94 [0.77 - 1.15] |  | 4582 (41.3%) | 0.99 [0.85 - 1.17] |  | 4582 (22.4%) | 1.10 [0.91 - 1.34] | |
|  | Trial round 5 |  | 2373 (64.8%) | 1.00 [0.80 - 1.25] |  | 2373 (44.0%) | 1.11 [0.93 - 1.33] |  | 2373 (19.9%) | 1.02 [0.82 - 1.28] | |
|  | Trial round 6 |  | 2043 (63.2%) | 0.97 [0.76 - 1.24] |  | 2043 (44.3%) | 1.28 [1.06 - 1.56] |  | 2043 (18.0%) | 0.84 [0.66 - 1.09] | |
|  | Trial round 7 |  | 438 (63.7%) | 0.75 [0.48 - 1.20] |  | 438 (42.7%) | 1.01 [0.70 - 1.45] |  | 438 (15.3%) | 0.81 [0.51 - 1.29] | |
| **Interaction Arm:Trial Round** | Intervention arm:round 2 | 0.20 | 2289 (63.5%) | 0.84 [0.67 - 1.05] | <0.001 | 2289 (45.3%) | 1.40 [1.17 - 1.69] | <0.001 | 2289 (21.3%) | 0.73 [0.58 - 0.91] | |
|  | Intervention arm:round 3 |  | 2187 (67.0%) | 1.10 [0.88 - 1.36] |  | 2187 (39.6%) | 1.02 [0.85 - 1.21] |  | 2187 (23.2%) | 1.03 [0.83 - 1.27] | |
|  | Intervention arm:round 4 |  | 2038 (63.4%) | 1.00 [0.81 - 1.23] |  | 2038 (40.3%) | 1.19 [1.00 - 1.41] |  | 2038 (21.3%) | 0.67 [0.54 - 0.82] | |
|  | Intervention arm:round 5 |  | 1086 (62.0%) | 0.87 [0.68 - 1.13] |  | 1086 (40.6%) | 1.05 [0.85 - 1.29] |  | 1086 (19.4%) | 0.65 [0.50 - 0.84] | |
|  | Intervention arm:round 6 |  | 968 (61.5%) | 0.88 [0.67 - 1.15] |  | 968 (38.1%) | 0.81 [0.65 - 1.02] |  | 968 (20.9%) | 1.03 [0.78 - 1.36] | |
|  | Intervention arm:round 7 |  | 260 (64.7%) | 1.29 [0.75 - 2.22] |  | 260 (42.7%) | 1.23 [0.80 - 1.90] |  | 260 (15%) | 0.74 [0.42 - 1.31] | |
| **Calendar Round** | Calendar round 1 | 0.0019 | 3794 (62.9%) | 1.01 [0.77 - 1.32] | <0.001 | 3794 (42.9%) | 1.08 [0.87 - 1.33] | <0.001 | 3794 (17.9%) | 0.98 [0.75 - 1.28] | |
|  | Calendar round 2 |  | 789 (63.3%) | 1.06 [0.84 - 1.33] |  | 789 (41.2%) | 1.51 [1.25 - 1.81] |  | 789 (19.3%) | 1.05 [0.83 - 1.32] | |
|  | Calendar round 3 |  | 1889 (66.7%) | 1.00 [0.80 - 1.24] |  | 1889 (53.4%) | 1.09 [0.92 - 1.29] |  | 1889 (19.2%) | 1.15 [0.92 - 1.42] | |
|  | Calendar round 4 |  | 2186 (66.2%) | 1.15 [0.97 - 1.36] |  | 2186 (46.9%) | 1.00 [0.87 - 1.15] |  | 2186 (18.2%) | 1.18 [1.00 - 1.39] | |
|  | Calendar round 5 |  | 5110 (67.5%) | 0.93 [0.81 - 1.08] |  | 5110 (41.5%) | 0.87 [0.77 - 0.98] |  | 5110 (22.7%) | 1.54 [1.34 - 1.78] | |
|  | Calendar round 6 |  | 5376 (63.7%) | 1.14 [0.97 - 1.34] |  | 5376 (41.3%) | 1.05 [0.92 - 1.19] |  | 5376 (23.7%) | 1.06 [0.90 - 1.24] | |

**Table S2.** Full multivariable model for each sexual behaviour indicator among men in the ANRS 12249 TasP trial (2012-2016) (2/3)

|  |  | **Condom use at last sex - Regular partner** | | | **Condom use at last sex – Casual partner** | | |
| --- | --- | --- | --- | --- | --- | --- | --- |
|  |  | **pval** | **n (%)** | **aOR 95CI** | **pval** | **n (%)** | **aOR 95CI** |
| **Arm** | Intervention | 0.22 | 4630 (41.7%) | 0.85 [0.71 - 1.02] | 0.028 | 2832 (55.9%) | 0.77 [0.6 - 0.97] |
| **Trial Round** | Trial round 2 | 0.0055 | 1752 (38.4%) | 0.94 [0.73 - 1.21] | 0.36 | 967 (55.8%) | 0.88 [0.63 - 1.24] |
|  | Trial round 3 |  | 2063 (43.7%) | 1.06 [0.86 - 1.30] |  | 1118 (59.3%) | 1.37 [1.02 - 1.86] |
|  | Trial round 4 |  | 1996 (43.0%) | 1.04 [0.82 - 1.32] |  | 1198 (58.7%) | 1.10 [0.79 - 1.54] |
|  | Trial round 5 |  | 1105 (40.1%) | 0.84 [0.64 - 1.10] |  | 570 (54.8%) | 0.85 [0.59 - 1.24] |
|  | Trial round 6 |  | 947 (42.4%) | 0.89 [0.66 - 1.20] |  | 424 (57.2%) | 0.96 [0.62 - 1.49] |
|  | Trial round 7 |  | 205 (33.5%) | 0.71 [0.38 - 1.32] |  | 79 (52.6%) | 1.01 [0.47 - 2.19] |
| **Interaction Arm:Trial Round** | Intervention arm:round 2 | <0.001 | 829 (37.1%) | 1.02 [0.78 - 1.33] | 0.008 | 453 (58.5%) | 1.55 [1.09 - 2.2] |
|  | Intervention arm:round 3 |  | 934 (47.0%) | 1.42 [1.11 - 1.81] |  | 605 (55.4%) | 0.86 [0.61 - 1.21] |
|  | Intervention arm:round 4 |  | 871 (42.8%) | 1.23 [0.96 - 1.57] |  | 520 (57.5%) | 1.14 [0.81 - 1.59] |
|  | Intervention arm:round 5 |  | 469 (46.1%) | 1.82 [1.35 - 2.46] |  | 264 (57.8%) | 1.68 [1.11 - 2.55] |
|  | Intervention arm:round 6 |  | 387 (46.2%) | 1.72 [1.25 - 2.36] |  | 228 (57.3%) | 1.41 [0.89 - 2.25] |
|  | Intervention arm:round 7 |  | 120 (36.4%) | 1.29 [0.64 - 2.56] |  | 44 (51.2%) | 1.05 [0.41 - 2.68] |
| **Calendar Round** | Calendar round 1 | 0.37 | 1712 (41.8%) | 0.86 [0.61 - 1.22] | 0.0065 | 767 (57.6%) | 0.76 [0.50 - 1.17] |
|  | Calendar round 2 |  | 376 (37.2%) | 0.97 [0.73 - 1.29] |  | 219 (48.8%) | 0.92 [0.63 - 1.34] |
|  | Calendar round 3 |  | 836 (39.5%) | 0.87 [0.67 - 1.14] |  | 342 (52.8%) | 0.97 [0.68 - 1.39] |
|  | Calendar round 4 |  | 624 (36.9%) | 0.96 [0.78 - 1.18] |  | 311 (56.1%) | 1.17 [0.87 - 1.56] |
|  | Calendar round 5 |  | 2268 (41.6%) | 0.98 [0.82 - 1.17] |  | 1424 (59.4%) | 0.93 [0.74 - 1.18] |
|  | Calendar round 6 |  | 2344 (41.1%) | 0.87 [0.72 - 1.04] |  | 1529 (57.1%) | 0.88 [0.67 - 1.15] |

**Table S2.** Full multivariable model for each sexual behaviour indicator among men in the ANRS 12249 TasP trial (2012-2016) (3/3)

|  |  | **≥ 2 sexual partnerships in the past 6 months** | | | **Concurrency (at the time of survey)** | | |
| --- | --- | --- | --- | --- | --- | --- | --- |
|  |  | **pval** | **n (%)** | **aOR 95CI** | **pval** | **n (%)** | **aOR 95CI** |
| **Arm** | Intervention | 0.46 | 11115 (8.6%) | 1.08 [0.88 - 1.31] | 0.58 | 11115 (8%) | 0.94 [0.75 - 1.17] |
| **Trial Round** | Trial round 2 | 0.68 | 5081 (9%) | 0.88 [0.67 - 1.17] | 0.8 | 5081 (8.7%) | 0.84 [0.59 - 1.18] |
|  | Trial round 3 |  | 4648 (7.7%) | 0.87 [0.67 - 1.13] |  | 4648 (7.4%) | 0.79 [0.58 - 1.06] |
|  | Trial round 4 |  | 4582 (6.3%) | 0.93 [0.70 - 1.24] |  | 4582 (5.7%) | 0.84 [0.60 - 1.17] |
|  | Trial round 5 |  | 2373 (6.6%) | 0.91 [0.65 - 1.27] |  | 2373 (6.5%) | 0.78 [0.53 - 1.14] |
|  | Trial round 6 |  | 2043 (5.6%) | 1.26 [0.87 - 1.83] |  | 2043 (5.4%) | 1.19 [0.77 - 1.82] |
|  | Trial round 7 |  | 438 (6.4%) | 1.57 [0.80 - 3.10] |  | 438 (4.4%) | 1.23 [0.52 - 2.92] |
| **Interaction Arm:Trial Round** | Intervention arm:round 2 | 0.53 | 2289 (9.7%) | 1.10 [0.81 - 1.48] | 0.26 | 2289 (9.4%) | 1.28 [0.90 - 1.81] |
|  | Intervention arm:round 3 |  | 2187 (8.9%) | 1.25 [0.92 - 1.69] |  | 2187 (8.8%) | 1.46 [1.03 - 2.07] |
|  | Intervention arm:round 4 |  | 2038 (6.8%) | 1.03 [0.75 - 1.40] |  | 2038 (6.1%) | 1.15 [0.8 0- 1.65] |
|  | Intervention arm:round 5 |  | 1086 (7.2%) | 1.10 [0.75 - 1.62] |  | 1086 (7.4%) | 1.40 [0.91 - 2.17] |
|  | Intervention arm:round 6 |  | 968 (5.1%) | 0.79 [0.51 - 1.22] |  | 968 (4.9%) | 0.91 [0.55 - 1.49] |
|  | Intervention arm:round 7 |  | 260 (6.2%) | 0.80 [0.35 - 1.80] |  | 260 (4%) | 0.75 [0.25 - 2.22] |
| **Calendar Round** | Calendar round 1 | <0.001 | 3794 (4.7%) | 4.20 [2.94 - 6.01] | <0.001 | 3794 (4.1%) | 3.74 [2.45 - 5.73] |
|  | Calendar round 2 |  | 789 (15.3%) | 3.69 [2.67 - 5.10] |  | 789 (13.7%) | 2.95 [2.01 - 4.32] |
|  | Calendar round 3 |  | 1889 (13.9%) | 3.39 [2.50 - 4.59] |  | 1889 (10.9%) | 3.33 [2.32 - 4.79] |
|  | Calendar round 4 |  | 2186 (13.2%) | 2.01 [1.53 - 2.63] |  | 2186 (11.6%) | 2.11 [1.53 - 2.90] |
|  | Calendar round 5 |  | 5110 (8.5%) | 1.99 [1.56 - 2.54] |  | 5110 (8.0%) | 2.16 [1.62 - 2.88] |
|  | Calendar round 6 |  | 5376 (7.6%) | 1.57 [1.21 - 2.04] |  | 5376 (7.4%) | 1.78 [1.31 - 2.42] |

**Table S3.** Full multivariable model for each sexual behaviour indicator among women in the ANRS 12249 TasP trial (2012-2016) (1/2)

|  |  | **Had a sexual intercourse (past month)** | | | **≥ 1 regular partner (past 6 months)** | | | **≥ 1 casual Partner (past 6 months)** | | |
| --- | --- | --- | --- | --- | --- | --- | --- | --- | --- | --- |
|  |  | **pval** | **n (%)** | **aOR 95CI** | **pval** | **n (%)** | **aOR 95CI** | **pval** | **n (%)** | **aOR 95CI** |
| **Arm** | Intervention | 0.099 | 25757 (48.7%) | 1.01 [0.92 - 1.11] | <0.001 | 25757 (38.4%) | 0.83 [0.77 - 0.91] | <0.001 | 25757 (13.6%) | 1.44 [1.27 - 1.64] |
| **Trial Round** | Trial round 2 | 0.02 | 11576 (50.3%) | 1.09 [0.94 - 1.25] | <0.001 | 11576 (42.1%) | 1.14 [1.01 - 1.29] | <0.001 | 11576 (13%) | 0.85 [0.70 - 1.02] |
|  | Trial round 3 |  | 10564 (48.7%) | 0.90 [0.80 - 1.02] |  | 10564 (38.8%) | 1.08 [0.98 - 1.19] |  | 10564 (12.3%) | 0.84 [0.71 - 0.99] |
|  | Trial round 4 |  | 10441 (48.2%) | 0.94 [0.83 - 1.07] |  | 10441 (38.5%) | 1.02 [0.91 - 1.13] |  | 10441 (14.5%) | 1.12 [0.95 - 1.32] |
|  | Trial round 5 |  | 5071 (49%) | 0.96 [0.83 - 1.10] |  | 5071 (40.5%) | 1.12 [0.99 - 1.27] |  | 5071 (11.5%) | 0.83 [0.69 - 1.01] |
|  | Trial round 6 |  | 4671 (45.8%) | 0.80 [0.69 - 0.93] |  | 4671 (39.2%) | 1.23 [1.08 - 1.40] |  | 4671 (11.1%) | 0.62 [0.50 - 0.76] |
|  | Trial round 7 |  | 1075 (49.7%) | 0.97 [0.75 - 1.26] |  | 1075 (37.6%) | 0.92 [0.73 - 1.16] |  | 1075 (9.0%) | 0.94 [0.65 - 1.36] |
| **Interaction Arm:Trial Round** | Intervention arm:round 2 | <0.001 | 5473 (47%) | 0.78 [0.67 - 0.91] | <0.001 | 5473 (42.4%) | 1.21 [1.07 - 1.37] | <0.001 | 5473 (13.6%) | 0.80 [0.66 - 0.96] |
|  | Intervention arm:round 3 |  | 5068 (49.3%) | 1.02 [0.89 - 1.17] |  | 5068 (35.5%) | 0.90 [0.80 - 1.01] |  | 5068 (14.8%) | 1.07 [0.89 - 1.28] |
|  | Intervention arm:round 4 |  | 4785 (46.8%) | 0.88 [0.77 - 1.01] |  | 4785 (38.6%) | 1.20 [1.06 - 1.35] |  | 4785 (13.4%) | 0.61 [0.51 - 0.72] |
|  | Intervention arm:round 5 |  | 2349 (48.9%) | 0.95 [0.81 - 1.13] |  | 2349 (38.9%) | 1.05 [0.91 - 1.22] |  | 2349 (11.5%) | 0.71 [0.57 - 0.88] |
|  | Intervention arm:round 6 |  | 2192 (47.7%) | 1.11 [0.93 - 1.31] |  | 2192 (35.3%) | 0.86 [0.75 - 1.00] |  | 2192 (13.9%) | 1.23 [0.98 - 1.55] |
|  | Intervention arm:round 7 |  | 614 (51.6%) | 1.05 [0.76 - 1.45] |  | 614 (38.9%) | 1.40 [1.06 - 1.84] |  | 614 (8.1%) | 0.47 [0.30 - 0.75] |
| **Calendar Round** | Calendar round 1 | <0.001 | 9173 (47.3%) | 0.97 [0.81 - 1.15] | <0.001 | 9173 (38.2%) | 1.20 [1.04 - 1.39] | <0.001 | 9173 (12.2%) | 0.67 [0.53 - 0.84] |
|  | Calendar round 2 |  | 1651 (49.6%) | 0.92 [0.79 - 1.06] |  | 1651 (39.9%) | 1.61 [1.42 - 1.82] |  | 1651 (10.5%) | 0.58 [0.47 - 0.71] |
|  | Calendar round 3 |  | 4643 (48.1%) | 1.08 [0.94 - 1.25] |  | 4643 (47.5%) | 1.23 [1.09 - 1.39] |  | 4643 (9.1%) | 0.60 [0.50 - 0.73] |
|  | Calendar round 4 |  | 5078 (51.2%) | 1.07 [0.96 - 1.18] |  | 5078 (43.9%) | 1.13 [1.03 - 1.24] |  | 5078 (8.1%) | 0.87 [0.76 - 0.99] |
|  | Calendar round 5 |  | 11263 (51.1%) | 1.01 [0.93 - 1.11] |  | 11263 (39.3%) | 0.97 [0.90 - 1.05] |  | 11263 (12.9%) | 1.21 [1.08 - 1.35] |
|  | Calendar round 6 |  | 11667 (49.2%) | 0.98 [0.89 - 1.08] |  | 11667 (39.6%) | 1.07 [0.99 - 1.17] |  | 11667 (14.8%) | 0.99 [0.87 - 1.13] |

**Table S3.** Full multivariable model for each sexual behaviour indicator among women in the ANRS 12249 TasP trial (2012-2016) (2/2)

|  |  | **Condom use at last sex - Regular partner** | | | **Condom use at last sex – Casual partner** | | |
| --- | --- | --- | --- | --- | --- | --- | --- |
|  |  | **pval** | **n (%)** | **aOR 95CI** | **pval** | **n (%)** | **aOR 95CI** |
| **Arm** | Intervention | 0.83 | 9211 (40.2%) | 0.92 [0.81 - 1.06] | 0.73 | 3289 (48.8%) | 0.88 [0.69 - 1.12] |
| **Trial Round** | Trial round 2 | <0.001 | 3307 (37.9%) | 1.22 [1.02 - 1.46] | <0.001 | 1040 (46.4%) | 1.10 [0.78 - 1.55] |
|  | Trial round 3 |  | 4030 (42.3%) | 1.20 [1.03 - 1.41] |  | 1297 (50.3%) | 1.62 [1.19 - 2.19] |
|  | Trial round 4 |  | 4022 (43.6%) | 1.23 [1.04 - 1.46] |  | 1528 (48.7%) | 1.33 [0.97 - 1.83] |
|  | Trial round 5 |  | 2057 (40.1%) | 0.95 [0.77 - 1.16] |  | 584 (54.6%) | 1.77 [1.23 - 2.55] |
|  | Trial round 6 |  | 1839 (42.3%) | 1.05 [0.84 - 1.31] |  | 524 (48.6%) | 1.98 [1.32 - 2.98] |
|  | Trial round 7 |  | 404 (30.6%) | 0.46 [0.30 - 0.71] |  | 97 (53.1%) | 1.58 [0.77 - 3.23] |
| **Interaction Arm:Trial Round** | Intervention arm:round 2 | <0.001 | 1594 (34.8%) | 0.82 [0.67 - 0.99] | 0.029 | 520 (49.5%) | 1.43 [1.01 - 2.03] |
|  | Intervention arm:round 3 |  | 1767 (43.8%) | 1.15 [0.97 - 1.37] |  | 743 (48.6%) | 0.95 [0.68 - 1.33] |
|  | Intervention arm:round 4 |  | 1850 (43.5%) | 1.09 [0.91 - 1.29] |  | 643 (50.8%) | 1.25 [0.91 - 1.73] |
|  | Intervention arm:round 5 |  | 915 (44.8%) | 1.54 [1.23 - 1.91] |  | 269 (57.3%) | 1.38 [0.91 - 2.10] |
|  | Intervention arm:round 6 |  | 779 (45.1%) | 1.31 [1.04 - 1.64] |  | 306 (44.3%) | 0.78 [0.51 - 1.21] |
|  | Intervention arm:round 7 |  | 239 (35.7%) | 2.14 [1.32 - 3.46] |  | 50 (59.2%) | 1.70 [0.70 - 4.17] |
| **Calendar Round** | Calendar round 1 | <0.001 | 3510 (42.5%) | 0.72 [0.55 - 0.94] | 0.0039 | 1122 (47.7%) | 1.02 [0.65 - 1.58] |
|  | Calendar round 2 |  | 662 (29.2%) | 0.90 [0.73 - 1.11] |  | 176 (36.6%) | 1.67 [1.14 - 2.43] |
|  | Calendar round 3 |  | 1614 (36.1%) | 0.73 [0.60 - 0.89] |  | 315 (50.3%) | 1.09 [0.74 - 1.59] |
|  | Calendar round 4 |  | 1043 (35.8%) | 0.97 [0.83 - 1.12] |  | 192 (45.3%) | 1.62 [1.25 - 2.09] |
|  | Calendar round 5 |  | 4437 (40.7%) | 0.90 [0.79 - 1.02] |  | 1474 (51.9%) | 1.10 [0.90 - 1.35] |
|  | Calendar round 6 |  | 4628 (40%) | 0.90 [0.79 - 1.02] |  | 1760 (48.2%) | 0.95 [0.75 - 1.21] |
